# Assessing the Variation within the Oral Microbiome of Healthy Adults

**DOI:** 10.1128/mSphere.00451-20

**Published:** 2020-09-30

**Authors:** Jacob T. Nearing, Vanessa DeClercq, Johan Van Limbergen, Morgan G. I. Langille

**Affiliations:** a Department of Microbiology and Immunology, Dalhousie University, Halifax, Nova Scotia, Canada; b Population Cancer Research Program, Dalhousie University, Halifax, Nova Scotia, Canada; c Division of Pediatric Gastroenterology & Nutrition, Emma Children’s Hospital, Amsterdam University Medical Centers, Amsterdam, The Netherlands; d Department of Pediatrics, Dalhousie University, Halifax, Nova Scotia, Canada; e Department of Pharmacology, Dalhousie University, Halifax, Nova Scotia, Canada; The Jackson Laboratory

**Keywords:** 16S rRNA, microbiome, oral microbiology

## Abstract

More than 1,000 different species of microbes have been found to live within the human oral cavity, where they play important roles in maintaining both oral and systemic health. Several studies have identified the core members of this microbial community; however, the factors that determine oral microbiome composition are not well understood. In this study, we exam the salivary oral microbiome of 1,049 Atlantic Canadians using 16S rRNA gene sequencing to determine which dietary, lifestyle, and anthropometric features play a role in shaping microbial community composition. Features that were identified as being significantly associated with overall composition then were additionally examined for genera, amplicon sequence variants, and predicted pathway abundances that were associated with these features. Several associations were replicated in an additional secondary validation data set. Overall, we found that several anthropometric measurements, including waist-hip ratio (WHR), height, and fat-free mass, as well as age and sex, were associated with overall oral microbiome structure in both our exploratory and validation data sets. We were unable to validate any dietary impacts on overall taxonomic oral microbiome composition but did find evidence to suggest potential contributions from factors such as the number of vegetable and refined grain servings an individual consumes. Interestingly, each one of these factors on its own was associated with only minor shifts in the overall taxonomic composition of the oral microbiome, suggesting that future biomarker identification for several diseases associated with the oral microbiome can be undertaken without the worry of confounding factors obscuring biological signals.

**IMPORTANCE** The human oral cavity is inhabited by a diverse community of microbes, known as the human oral microbiome. These microbes play a role in maintaining both oral and systemic health and, as such, have been proposed to be useful biomarkers of disease. However, to identify these biomarkers, we first need to determine the composition and variation of the healthy oral microbiome. In this report, we investigate the oral microbiome of 1,049 healthy individuals to determine which genera and amplicon sequence variants are commonly found between individual oral microbiomes. We then further investigate how lifestyle, anthropometric, and dietary choices impact overall microbiome composition. Interestingly, the results from this investigation showed that while many features were significantly associated with oral microbiome composition, no single biological factor explained a variation larger than 2%. These results indicate that future work on biomarker detection may be encouraged by the lack of strong confounding factors.

## Abstract (precis)

The human oral cavity is inhabited by a diverse community of microbes, known as the human oral microbiome. These microbes play a role in maintaining both oral and systemic health and, as such, have been proposed to be useful biomarkers of disease. However, to identify these biomarkers, we first need to determine the composition and variation of the healthy oral microbiome. In this report, we investigate the oral microbiome of 1,049 healthy individuals to determine which genera and amplicon sequence variants are commonly found between individual oral microbiomes. We then further investigate how lifestyle, anthropometric, and dietary choices impact overall microbiome composition. Interestingly, the results from this investigation showed that while many features were significantly associated with oral microbiome composition, no single biological factor explained a variation larger than 2%. These results indicate that future work on biomarker detection may be encouraged by the lack of strong confounding factors.

## INTRODUCTION

The human oral cavity is colonized by numerous bacteria, fungi, viruses, and archaea that make a rich microbial community known as the oral microbiome. This microbial community is one of the most diverse sites of microbial growth within the human body; only the colon houses a more diverse consortia of microbes (1). To date, more than 1,000 different bacterial species have been found to colonize the oral cavity (2) on various surfaces, including the tongue, teeth, cheek, and gingivae (1). These communities of microbes are responsible for various functions that can both maintain and deplete oral health. For example, the presence of biofilms containing bacterial species such as Streptococcus mutans and other aciduric bacteria can damage hard dental surfaces and lead to dental caries (3, 4). Furthermore, the oral microbiome is known to play a role in a myriad of other oral diseases, including oral cancer (5), periodontitis (6, 7), and gingivitis (8, 9). In addition to well-established associations between oral and cardiac health (10), recent work has also begun to show that the oral microbiome may play a role in the health of other distal sites within the human body. For example, the enrichment of both Porphyromonas gingivalis and Aggregatibacter actinomycetemcomitans has been associated with a higher risk of pancreatic cancer (11). Furthermore, several oral bacteria, including *Streptococcus* and *Prevotella* species, have been found to be in higher relative abundance among individuals with colorectal cancer (12). Other than these two cancers, a number of other distal diseases have been associated with oral microbiome composition, including prostate cancer (13) and inflammatory bowel disease (14).

Due to the associations between these diseases and the oral microbiome, its composition has been proposed as a useful biomarker for human health and disease. With this in mind, various studies have attempted to identify core members of the “healthy” oral microbiome (1, 15–18) to aid in disease detection. These studies have uncovered that, at the genus level, the oral microbiome remains relatively stable between individuals (1, 18) and across multiple geographic locations (16, 19), but at deeper taxonomic resolutions it can be variable. This indicates that other factors, such as dietary, anthropometric, or sociodemographic factors, may play a role in shaping the oral microbiome (15, 17, 20–23). Various studies have focused on individual factors that may cause shifts in the oral microbiome, such as ethnicity (1, 23), alcohol consumption (24), smoking (25), obesity (26, 27), and dietary patterns (28). However, to date, only a small number of studies have looked at the relative contributions of each of these factors to oral microbiome variability in a single cohort. Takeshita et al. examined the oral microbiome of 2,343 adults living in Japan using 16S rRNA gene sequencing and identified that higher abundances of *Prevotella* and *Veillonella* species were associated with old age, higher body mass index (BMI), and poor overall oral health (17). Another study by Renson et al. in adults living in New York City also found that variation in taxonomic abundances could be linked to marital status, ethnicity, education, and age (21). Further, work by Belstrøm et al. examined the oral microbiome of 292 Danish individuals with low levels of dental caries and periodontitis using microarrays and found that while socioeconomic status impacted oral microbiome profiles, diet, BMI, age, and sex had no statistical impact on microbial abundances (20). This study, however, was only able to identify the abundances of taxa that had a corresponding probe, which could explain its disagreement with other work. Overall, these studies have indicated that biological differences, such as sex and BMI, as well as lifestyle and sociodemographic differences can impact oral microbiome composition.

While these studies have shed light on the variation of the oral microbiome, it is currently unclear to what extent these factors play a role in shaping the oral microbiome of an individual. Without identifying the effect size of each of these factors relative to one another, it is difficult to identify the correct variables that should be controlled for in case-control studies of the oral microbiome. Furthermore, each of these studies has identified different taxa that are impacted by various factors, such as sex, BMI, and age. This could be due to many factors, including systemic bias introduced via the use of different sequencing or bioinformatic protocols/tools (29) or differences in the studied cohorts. Therefore, the identification of microbes that are impacted by factors such as sex, BMI, or diet could help identify potential interactions between the oral microbiome, health, and disease.

**FIG 1:**
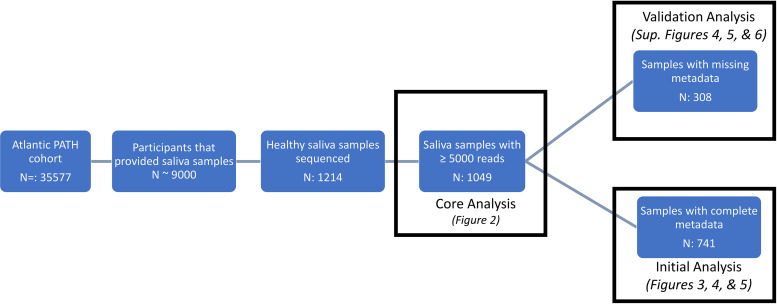
Flowchart of sample selection from the Atlantic Partnership for Tomorrow’s Health cohort. A total of 35,577 individuals participated in the Atlantic Partnership for Tomorrow’s Health cohort, and ∼9,000 individuals provided saliva samples. Of those, a subset of 1,214 saliva samples from healthy individuals underwent 16S rRNA gene sequencing. Samples below 5,000 reads were filtered out, and two data sets were created for discovery and validation analysis.

Here, we report the variation within the healthy oral microbiome by examining 741 samples from nonsmoking healthy individuals living within the Atlantic provinces of Canada. We then validated our results on a smaller subset of individuals (*n* = 308) from the same cohort (Fig. 1). The bacterial oral microbiome composition of these individuals was investigated through 16S rRNA gene sequencing from saliva samples provided by each participant. Compositions were then compared using 41 different variables, including anthropometric, dietary, and sociodemographic factors (Table 1). In this investigation, we determined which of these factors play a role in shaping the oral microbiome and to what extent these factors can explain the overall oral microbiome composition.

**TABLE 1:** Cohort characteristic and variables analyzed for oral microbiome composition

Parameter^*a*^	Overall value
No. of participants	1,214
Rural/urban [no. (%)]	
Urban	1,050 (86.5)
Rural	126 (10.4)
NA	38 (3.1)
Province [no. (%)]	
New Brunswick	124 (10.2)
Nova Scotia	1,070 (88.1)
Prince Edward Island	16 (1.3)
NA	Data repressed
Economic region (no.)	
Annapolis Valley	52
Cape Breton	142
Edmundston–Woodstock	Data repressed
Fredericton–Oromocto	44
Halifax	773
Moncton–Richibucto	32
North Shore	41
Prince Edward Island	16
Saint John–St. Stephen	45
Southern Shore	28
Sex [no. (%)]	
Female	846 (69.7)
Male	368 (30.3)
BMI [mean (SD)]	27.30 (4.55)
Waist size [cm; mean (SD)]	90.96 (12.79)
Hip size [cm; mean (SD)]	104.29 (9.45)
Waist-hip ratio [mean (SD)]	0.87 (0.08)
Height (cm; mean [SD])	167.06 (8.90)
Weight (kg; mean [SD])	76.39 (14.99)
Age (yr; mean [SD])	55.39 (7.80)
Fat mass [kg; mean (SD)]	25.26 (9.55)
Fat-free mass [kg; mean (SD)]	51.05 (10.87)
Body fat percentage [mean (SD)]	32.68 (8.61)
Vegetable servings [mean (SD)]	2.56 (1.98)
Fruit servings [mean (SD)]	2.00 (1.45)
Juice servings [mean (SD)]	0.69 (0.95)
Whole grain servings [mean (SD)]	2.11 (1.43)
Refined grain servings [mean (SD)]	0.67 (0.86)
Milk product servings [mean (SD)]	2.04 (1.29)
Egg servings per wk [mean (SD)]	3.25 (2.68)
Meat/poultry servings [mean (SD)]	1.53 (1.35)
Fish servings [mean (SD)]	0.51 (0.67)
Tofu servings [mean (SD)]	0.04 (0.18)
Bean servings [mean (SD)]	0.36 (0.55)
Nut/seed servings [mean (SD)]	0.69 (0.68)
Dessert frequency [no. (%)]	
Never	109 (9.0)
>1 time a mo	153 (12.6)
∼1 time a mo	228 (18.8)
2–3 times a mo	173 (14.3)
1 time a wk	85 (7.0)
2–3 times a wk	115 (9.5)
4–5 times a wk	58 (4.8)
6–7 times a wk	169 (13.9)
NA	124 (10.2)
Avoidance of particular foods [no. (%)]	
Never	853 (70.3)
Often	11 (0.9)
Prefer not to answer	15 (1.2)
Rarely	163 (13.4)
Sometimes	52 (4.3)
NA	120 (9.9)
Oil on bread [no. (%)]	
Butter	371 (30.6)
Low-fat margarine	272 (22.4)
Full-fat margarine	300 (24.7)
None	109 (9.0)
Olive oil	36 (3.0)
NA	126 (10.4)
Artificial sweeteners [no. (%)]	
Almost never	976 (80.4)
About 1/4 of the time	24 (2.0)
About 1/2 of the time	16 (1.3)
About 3/4 of the time	12 (1.0)
Almost always or always	53 (4.4)
NA	133 (11.0)
Nondiet soda frequency [no. (%)]	
0 days a wk	432 (35.6)
1–3 days per mo	459 (37.8)
1–5 days a wk	167 (13.8)
6–7 days a wk	27 (2.2)
NA	129 (10.6)
Diet sugar drink frequency [no. (%)]	
0 days a wk	513 (42.3)
1–3 days per mo	356 (29.3)
1–5 days a wk	156 (12.9)
6–7 days a wk	57 (4.7)
NA	132 (10.9)
Soy/fish sauce usage [no. (%)]	
Never at the table	424 (34.9)
Rarely at the table	441 (36.3)
Sometimes at the table	217 (17.9)
At most meals of eating occasions	9 (0.7)
NA	123 (10.1)
Salt seasoning [no. (%)]	
Never	368 (30.3)
Rarely	347 (28.6)
Sometimes	219 (18.0)
Most meals	157 (12.9)
NA	123 (10.1)
Fast food frequency [no. (%)]	
Never	149 (12.3)
>1 time per mo	384 (31.6)
1–3 times per mo	366 (30.1)
1–6 times per wk	191 (15.7)
1 or more times per day	Data repressed
NA	122 (10.0)
Alcohol frequency [no. (%)]	
Never	61 (5.0)
>1 time a mo	192 (15.8)
∼1 time a mo	70 (5.8)
2–3 times a mo	171 (14.1)
1 time a wk	170 (14.0)
2–3 times a wk	259 (21.3)
4–5 times a wk	127 (10.5)
6–7 times a wk	112 (9.2)
NA	52 (4.3)
Education level [no. (%)]	
High school or below	208 (17.1)
Non-Bachelors postsecondary	425 (35.0)
Bachelors	334 (27.5)
Graduate	242 (19.9)
NA	Data repressed
Income [no. (%)]	
Below $25,000 CAD	41 (3.4)
$25,000–$49,999 CAD	157 (12.9)
$50,000–$74,999 CAD	244 (20.1)
$75,000–$99,999 CAD	244 (20.1)
$100,000–$149,999 CAD	291 (24.0)
Greater than $150,000 CAD	179 (14.7)
NA	58 (4.8)
Sleeping trouble frequency [no. (%)]	
None	104 (8.6)
Rarely	411 (33.9)
Some of the time	507 (41.8)
Most of the time	161 (13.3)
All the time	25 (2.1)
NA	Data repressed
Last dental visit [no. (%)]	
>6 mo ago	851 (70.1)
6 mo to >1 yr ago	221 (18.2)
1 yr to >2 yr ago	56 (4.6)
2 yrs to >3 yr ago	17 (1.4)
3 or more yr ago	24 (2.0)
NA	45 (3.7)
Sleeping light exposure [no. (%)]	
Virtually no light	561 (46.2)
Some light	613 (50.5)
A lot of light	36 (3.0)
NA	Data repressed
DNA extraction batch [no. (%)]	
Extraction.1	85 (7.0)
Extraction.10	66 (5.4)
Extraction.11	80 (6.6)
Extraction.12	78 (6.4)
Extraction.13	85 (7.0)
Extraction.14	57 (4.7)
Extraction.15	79 (6.5)
Extraction.16	0 (0.0)
Extraction.17	67 (5.5)
Extraction.2	85 (7.0)
Extraction.3	81 (6.7)
Extraction.4	68 (5.6)
Extraction.5	85 (7.0)
Extraction.6	92 (7.6)
Extraction.7	85 (7.0)
Extraction.8	60 (4.9)
Extraction.9	61 (5.0)

a NA represents responses of prefer not to answer or missing data. CAD, Canadian dollars.

## RESULTS

### The healthy oral microbiome is stable at the genus level but variable at higher resolutions.

**FIG 2:**
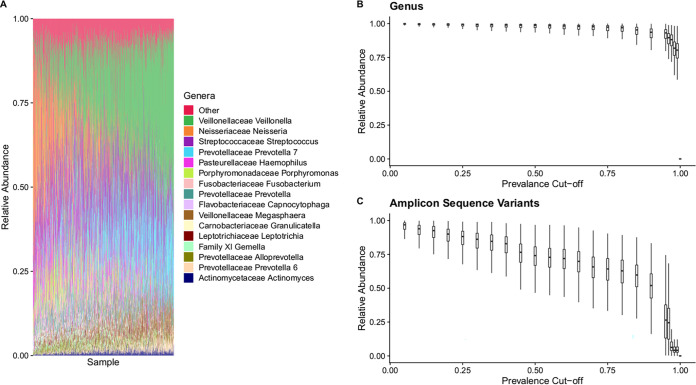
Atlantic Canadian oral microbiome composition is dominated by the genus *Veillonella* and is relatively similar at the genus level but highly variable at the ASV level. Samples were from the Atlantic Partnership for Tomorrow’s Health project (*n* = 1,049). Samples were subsampled to a depth of 5,000 reads. (A) Genera that had a mean relative abundance of less than 1% were grouped into “Other.” (B) Genera were removed at different sample presence cutoffs, and the remaining total mean relative abundance of nonfiltered genera was then calculated. (C) ASVs were removed at different sample presence cutoffs, and the remaining total mean relative abundance of nonfiltered ASVs was then calculated.

We examined the oral microbiome composition of the overall cohort containing 1,049 healthy individuals (Fig. 1) from Atlantic Canada to understand how anthropometric, sociodemographic, and dietary choices could alter oral microbiome composition. We found that 16 genera were found to have a mean relative abundance greater than 1% (Fig. 2A), with *Veillonella* having the largest mean contribution (21.49% ± 0.38%), followed by *Neisseria* (13.04% ± 0.40%), *Streptococcus* (11.86% ± 0.26%), and *Prevotella* 7 (11.55% ± 0.24%).

To characterize the core relative abundance of core genera and amplicon sequence variants (ASVs) within the oral microbiome of these samples, the mean relative abundance of genera/ASVs that were present in greater than a specific percentage of samples was analyzed. Interestingly, we found that at the genus level the oral microbiome is relatively stable, with 11 genera (see Fig. S2A and Table S1 in the supplemental material) present in greater than 99% of all individuals, making up, on average, a total relative abundance of 77.82% (Fig. 2B). However, this was not the case when we examined composition at a higher taxonomic resolution. We then found that only 5.17%, on average, of the total relative abundance of the oral microbiome was made up of 3 ASVs (Fig. S2B) shared between 99% of all participants in the study (Fig. 2C). These ASVs were classified as being in the *Granulicatella*, *Streptococcus*, and *Gemella* genera but could not confidently be assigned to a specific species.

10.1128/mSphere.00451-20.1 FIG S1 Rarefaction curve of all 1,049 Atlantic Partnership for Tomorrow’s Health saliva samples. Samples were divided by DNA extraction run to ensure that no large issues with DNA extraction occurred during sample preparation. All samples began plateauing at around 5,000 reads per sample. Download FIG S1, TIF file, 2.5 MB. Copyright © 2020 Nearing et al. 2020 Nearing et al. This content is distributed under the terms of the Creative Commons Attribution 4.0 International license.

10.1128/mSphere.00451-20.2 FIG S2 Number of genera and ASVs that passed a specific sample presence cutoff. (A) Genera were removed at different sample presence cutoffs, and the remaining number of genera was totaled. (B) ASVs were removed at different sample presence cutoffs, and the remaining number of ASVs was totaled. Download FIG S2, TIF file, 0.6 MB. Copyright © 2020 Nearing et al. 2020 Nearing et al. This content is distributed under the terms of the Creative Commons Attribution 4.0 International license.

### Demographic, anthropometric, and lifestyle choices have small but significant impacts on oral microbiome composition.

**FIG 3:**
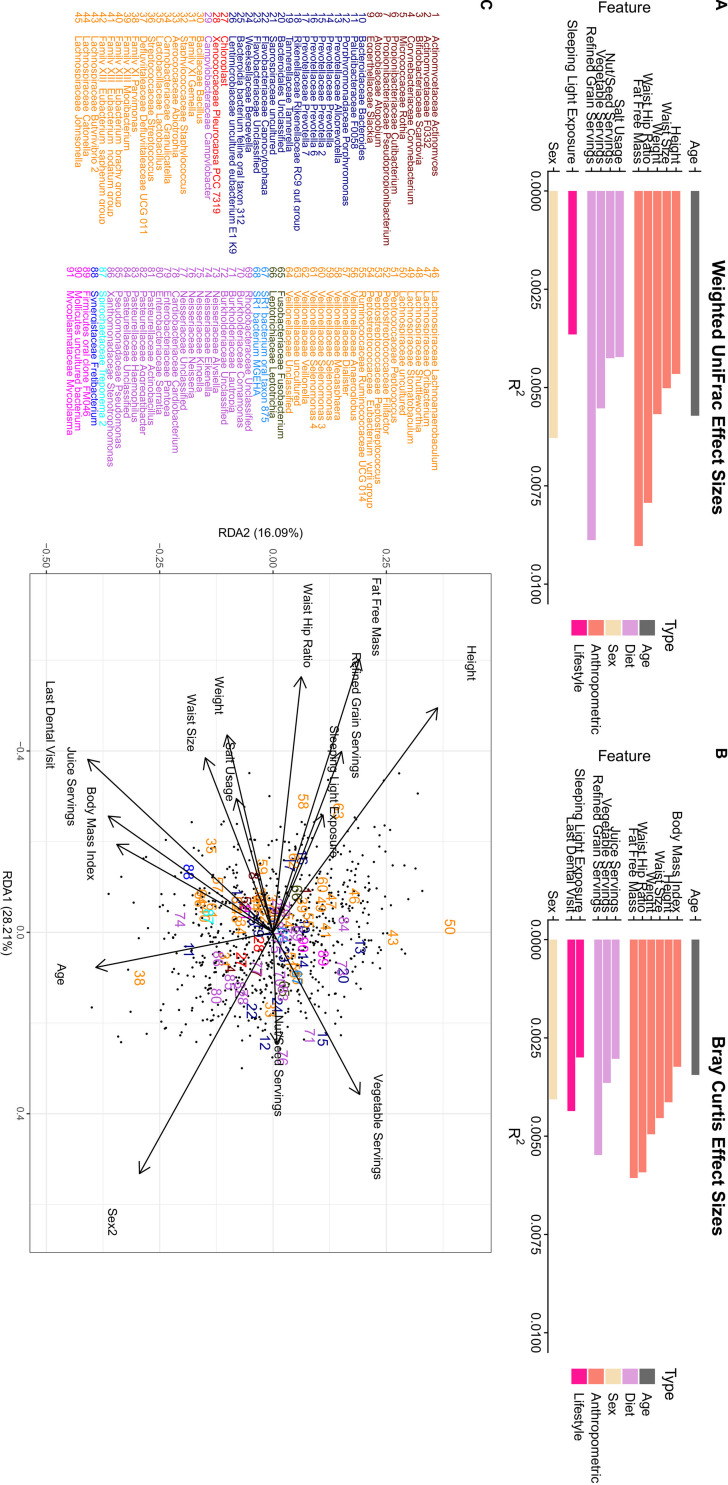
Various anthropometric, dietary, and lifestyle features are significantly associated with oral microbiome composition. Saliva samples were from the Atlantic Partnership for Tomorrow’s Health cohort (*n* = 741). Samples were subsampled to a depth of 5,000 reads. Two different metrics measuring beta diversity were tested, weighted Unifrac distances (A) and Bray-Curtis dissimilarity (B), using a PERMANOVA test while controlling for differences in DNA extraction and correction for false discovery (*q* < 0.1). Relationships between significant features, samples, and genera that were present in at least 10% of samples were then visualized by redundancy analysis (RDA) on center-log-ratio genus count tables. (C) Genera are colored by phylum and labeled numerically.

We examined the relationship of both alpha and beta diversities of the oral microbiome between 41 different variables that described various demographic, lifestyle, and anthropometric measures (Table 1). Samples were split into two different cohorts based on whether the subjects had answered all 41 variables of interest. A total of 741 individuals answered all 41 variables and were included in the exploratory cohort. From this cohort, we did not find any significant associations between any of the 41 variables tested and four different alpha diversity metrics (Faith’s phylogenetic diversity, number of ASVs, Shannon diversity, and evenness) after correction for multiple testing using linear models that were adjusted for DNA extraction batch (Data Set S1). We did, however, find 10 variables that were associated with differences in beta diversity as measured by both weighted UniFrac (Fig. 3A) and Bray-Curtis dissimilarity (Fig. 3B) (*q* < 0.1 by permutational multivariate analysis of variance [PERMANOVA]) (Data Set S1). We found two additional variables that were only associated with weighted UniFrac distances and three additional variables that were only associated with Bray-Curtis dissimilarity (*q* < 0.1 by PERMANOVA). Redundancy analysis (*P* = 0.001 by analysis of variance [ANOVA]) revealed that multiple anthropometric measures, such as height, fat-free mass, refined grain servings, sleeping light exposure, and waist-to-hip ratio were associated in similar manners. Furthermore, as expected, increases in all of these features were inversely associated with being female (Fig. 3C). As sex plays an important role in determining the height, fat-free mass, and waist-hip ratio of an individual, we attempted to determine whether sex was confounding our results from these variables. A separate analysis on weighted UniFrac distances controlling for sex indicated that fat-free mass (*P* = 0.02, *R*^2^ = 0.0039) and waist-hip ratio (*P* = 0.03, *R*^2^ = 0.0039), but not height (*P* = 0.44, *R*^2^ = 0.0012), were significantly associated with microbial composition despite differences in sex.

10.1128/mSphere.00451-20.10 DATA SET S1 Comparison of oral microbiome alpha diversity and beta diversity to various anthropometric, age, sex, lifestyle, and dietary features. This file contains all of the raw results from the comparison of four different alpha diversity metrics (Faith’s PD, richness, evenness, and Shannon diversity) and 2 different beta diversity metrics against 41 different metadata variables of interest. For alpha diversity analysis, each variable was tested using linear models while controlling for DNA extraction batch. Beta diversity analysis was done using a PERMANOVA test (Adonis function in vegan) while controlling for DNA extraction batch. Download Data Set S1, XLS file, 0.04 MB. Copyright © 2020 Nearing et al. 2020 Nearing et al. This content is distributed under the terms of the Creative Commons Attribution 4.0 International license.

Examining the amount of variation explained by each metadata feature by itself after controlling for DNA extraction showed small effect sizes for both weighted UniFrac distances and Bray-Curtis dissimilarities (*R*^2^ = 0.0030 to 0.009) (Fig. 3A and B). Of the features that were significant, sleeping light exposure explained the least amount of variation in both weighted UniFrac distances (*R*^2^ = 0.0036) and Bray-Curtis dissimilarity (*R*^2^ = 0.0030). We also found that fat-free mass explained the largest amount of variation in both weighted UniFrac (*R*^2^ = 0.009) and Bray-Curtis dissimilarity (*R*^2^ = 0.006). In general, we found that the rankings of effect sizes between these two different metrics agreed (Fig. 3A and B).

We also examined random forest machine learning classification and regression performance for each of these significant features. We found that, overall, random forest models performed poorly but did show slight associations between some variables (Fig. S3). For example, the area under the receiver operating curve (AUROC) for sex classification was 0.638, indicating slightly better than random performance. Regression models for features such as height and age showed an *R*^2^ of 0.10 and 0.075 with a root mean standard errors of 8.629 cm and 7.635 years, respectively (Fig. S3). Interestingly, some features performed extremely poorly, such as the number of refined grain servings (*R*^2^ = 8.22E−6) or vegetable servings (*R*^2^ = 0.004) (Fig. S3).

10.1128/mSphere.00451-20.3 FIG S3 Random forest machine learning performance on features that were significantly associated with overall oral microbiome composition. Random forest classification and regression models were trained for each feature found to be significantly associated with overall microbiome composition. Models were trained on ASV relative abundance tables using the initial discovery dataset (*N* = 741). Model performance was then validated on the smaller validation dataset (*N* = 308) using AUROC (A) and *R*^2^ (B). Download FIG S3, TIF file, 0.8 MB. Copyright © 2020 Nearing et al. 2020 Nearing et al. This content is distributed under the terms of the Creative Commons Attribution 4.0 International license.

Examining each significant factor in our weighted UniFrac analysis using a backward-selected multivariate PERMANOVA, we found that 7.0% of total oral microbiome variation could be explained by a total of 6 significant factors, including DNA extraction batch, despite using the same protocol, equipment, and personnel for each round (Table S2). Interestingly, of these 6 factors, DNA extraction number explained a considerable amount of the variation alone (4.18%) (Table S2). We found similar results examining beta diversity variation using Bray-Curtis dissimilarity with a slightly higher number of significant features and lower total variation explained (5.87%) (Table S3). It should be noted that many features were highly correlated with one another (*R* > 0.7), and, as such, model selection for these multivariate PERMANOVAs could have suffered due to the collinearity of these features. However, a model containing all features that were significantly associated with either weighted UniFrac or Bray-Curtis dissimilarity during univariate testing explained a similar level of variation for both weighted UniFrac and Bray-Curtis dissimilarity profiles (8.09% and 6.81%).

10.1128/mSphere.00451-20.8 TABLE S2 Multivariate weighted UniFrac PERMANOVA values. Features that were found to be significantly related to weighted UniFrac distances after univariate analysis (PERMANOVA) were then combined into a single multivariate PERMANOVA model. Features within this model were selected using a backward elimination stepwise regression strategy until all features were significant based on marginal *P* values. The total variation explained by this model (*R*^2^) was then analyzed to determine the total amount of oral microbiome variation that could be explained. Download Table S2, DOCX file, 0.01 MB. Copyright © 2020 Nearing et al. 2020 Nearing et al. This content is distributed under the terms of the Creative Commons Attribution 4.0 International license.

10.1128/mSphere.00451-20.9 TABLE S3 Multivariate Bray-Curtis PERMANOVA values. Features that were found to be significantly related to Bray-Curtis dissimilarities after univariate analysis (PERMANOVA) were then combined into a single multivariate PERMANOVA model. Features within this model were selected using a backward elimination stepwise regression strategy until all features were significant based on marginal *P* values. The total variation explained by this model (*R*^2^) was then analyzed to determine the total amount of oral microbiome variation that could be explained. Download Table S3, DOCX file, 0.01 MB. Copyright © 2020 Nearing et al. 2020 Nearing et al. This content is distributed under the terms of the Creative Commons Attribution 4.0 International license.

Redundancy analysis revealed several potential taxonomic associations with various features (*P* = 0.001 by ANOVA). For example, results for the genus *Megasphaera* (label 58, Fig. 3C) are in the same direction as those for increasing fat-free mass, height, waist-hip ratio, and daily refined grain servings but in the opposite direction of being female (Fig. 3C). Another uncultured genus in the *Veillonellaceae* family (label 63) was similarly grouped. The genus *Parvimonas* (label 38) is in a direction similar to that of increasing age and being female. Both *Lautropia* (label 71) and *Prevotella* 2 (label 15) are associated with increasing vegetable intake, and *Neisseria* (label 76) is associated with increasing nut/seed servings and decreasing refined grain servings (Fig. 3C). The only genus in the phylum *Synergistetes* that passed the 10% prevalence filtering was found to be associated with increasing juice servings, BMI, and time since last dental appointment. Overall, we found that phyla tended to cluster together, with *Firmicutes* and *Proteobacteria* clustering in opposite directions (Fig. 3C).

To help validate the associations we found between features and weighted UniFrac and Bray-Curtis dissimilarities, we analyzed an additional 308 samples from a smaller subset of the Atlantic Partnership for Tomorrow’s Health (PATH) cohort that had not completely answered all 41 variables of interest. We found that associations between both beta diversity metrics (weighted UniFrac and Bray-Curtis dissimilarity) and anthropometric features, such as height, weight, waist-hip ratio, and fat-free mass, were recoverable within our smaller cohort (Table 2 and Fig. S4). We were unable to recover any significant taxonomic dietary associations within this smaller validation cohort. We also were unable to recover taxonomic associations between lifestyle variables, such as sleeping light exposure or the time since an individual’s last dental visit. The inability to recover these differences could have been due to the highly reduced sample size within this validation cohort.

**TABLE 2:** Validation of beta diversity results

Metric and feature	*P* value	*R* ^2^
Weighted UniFrac		
Waist-hip ratio	0.0190	0.0116
Height	0.001	0.0117
Weight	0.010	0.0102
Fat-free mass	0.002	0.0172
Sex	0.0390	0.0080
Age	0.0120	0.0105
Bray-Curtis		
Waist-hip ratio	0.0140	0.0072
Height	0.0030	0.0118
Weight	0.0020	0.0096
Fat-free mass	0.0040	0.0110
Waist size	0.0210	0.0065
Age	0.0020	0.0106
Sex	0.0380	0.0059

10.1128/mSphere.00451-20.4 FIG S4 Anthropometric measurements, age, and sex are recoverable variables within a smaller validation dataset. Saliva samples from a smaller validation dataset from Atlantic Partnership for Tomorrow’s Health (*n* = 308) were examined by 16S rRNA gene sequencing. Previously found relationships in the original dataset were then tested within this data set to identify relationships that were robust to sample choice (*P* < 0.05). Samples were subsampled to a depth of 5,000 reads. Two different metrics were tested by weighted Unifrac distances (A) and Bray-Curtis dissimilarity (B) using a PERMANOVA test while controlling for differences in DNA extraction. Features were grouped based on type and ordered within their group based on effect size (*R*^2^). Download FIG S4, TIF file, 0.9 MB. Copyright © 2020 Nearing et al. 2020 Nearing et al. This content is distributed under the terms of the Creative Commons Attribution 4.0 International license.

### The abundance of various oral bacterial genera and ASVs are associated with anthropometric measurements and dietary choices in healthy individuals.

**FIG 4:**
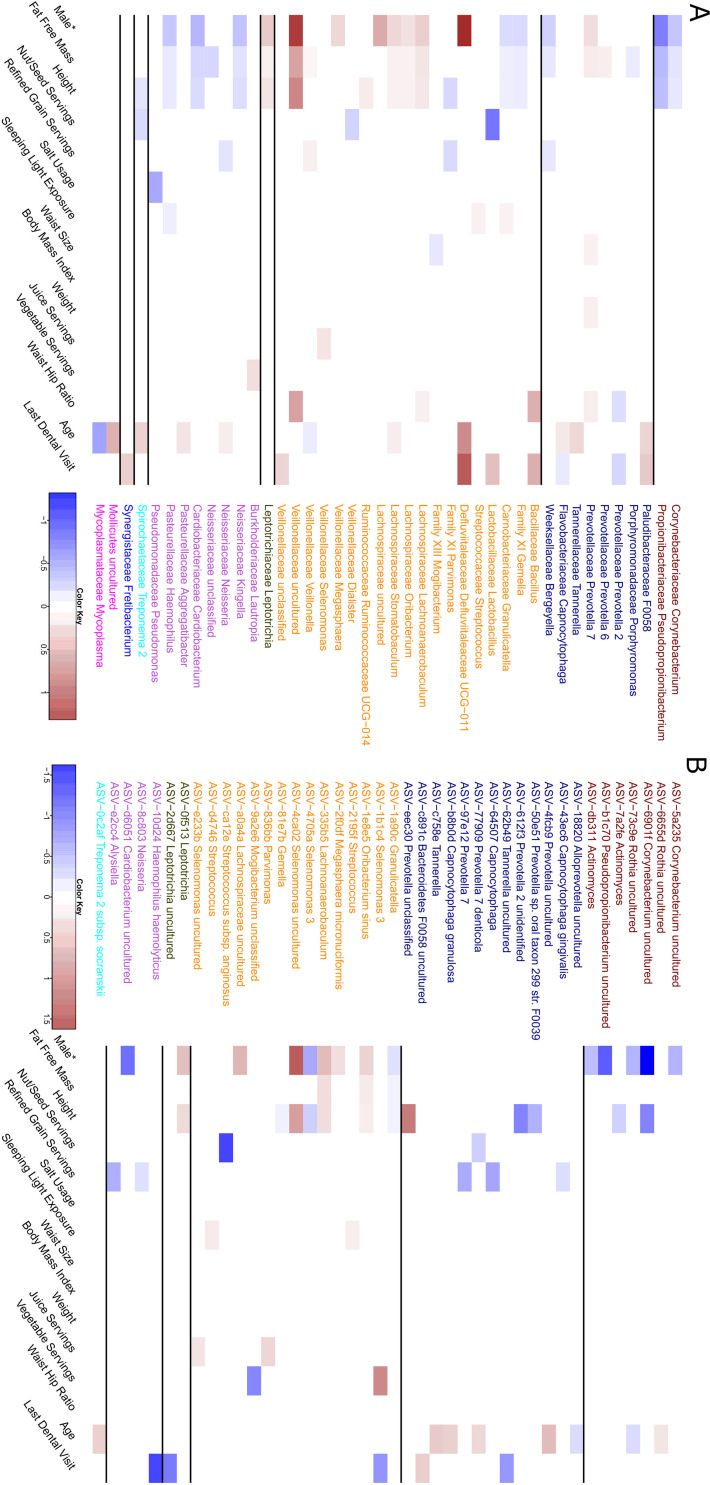
Differentially abundant genera and ASVs whose abundance profiles are associated with features found to influence oral microbiome composition. Genera (A) and ASVs (B) meeting a false discovery rate of *q* < 0.1 using the Corncob R package, which uses beta-binomial regressions. Each feature’s false discovery rate was corrected separately, and each was tested to control for differences in DNA extraction and differential variability within that feature. Ordinal variables were converted into a ranked scale for testing, and all features except for sex were scaled. The asterisk indicates that sex was treated as a categorical value; therefore, the magnitude is not directly comparable to other log odd ratios.

We next decided to identify genera that were associated with the 15 features previously identified as being associated with beta diversity in either the weighted UniFrac or Bray-Curtis dissimilarity analysis. We found 42 genera (Fig. 4A) and 42 ASVs (Fig. 4B) that had abundance profiles that were significantly associated with at least one of these features after controlling for DNA extraction. We found that sex, height, and fat-free mass shared similar genera and ASV associations. To control for the possibility of sex confounding our height and fat-free mass associations, we reanalyzed the data controlling for sex. We found that no ASVs or genera were significantly associated with fat-free mass after controlling for sex, and only 3 genera, “Chloroplast,” “unclassified *Burkholderiaceae*,” and *Treponema* 2, were significantly associated with height. Interestingly, two of these three genera were not previously associated with height in our initial analysis. These results suggest that many of these features associated with height or fat-free mass are driven by differences in sex. To test this, we also tested for differences in sex while controlling for both fat-free mass and height. Interestingly, we did not find any significantly associated ASVs and only three significantly associated genera, “*Defluvittaleaceae* UCG 011,” *Leptotrichia*, and *Treponema* 2.

We did not find any other features that shared similar patterns of taxonomic associations, but there were multiple genera with multiple feature associations. The genus *Prevotella* 7 had the highest number of features (5) associated with its relative abundance, including four anthropometric measurements (height, fat-free mass, waist size, waist-hip ratio, and weight) and sex. Interestingly, BMI was not significantly associated with any genera or ASVs despite many other anthropometric measures showing strong taxonomic signals. We were unable to identify any single ASVs associated with waist size and weight but were able to identify a small number of genera, including *Prevotella* 7, which was related to both, and *Mogibacterium*, which was associated with waist size. We also found that for some phyla, many taxa with significant associations had the same effect size direction. For example, genera in the *Actinobacteria* or *Proteobacteria* phyla tended to be negatively associated with fat-free mass, height, and being male. We also found several genera in the *Proteobacteria* phylum that were significantly associated with the amount of time since an individual’s last dental appointment.

In contrast, examining the ASVs associated with each feature, we found that in a small number of cases ASVs in the same genera had opposite directions of association to the same features. For example, two ASVs classified as uncultured *Rothia* both were significantly associated with age but in opposite directions, suggesting that lower taxonomic resolution is required to identify some associations. Furthermore, we also identified cases were ASVs that were associated with a feature were classified in a genus that was found not to be related to that feature. For example, uncultured *Selenomonas* ASV-4ca02 was strongly associated with being male, even though this entire collective genus was not (Fig. 4). Further examples include ASV-e2cc4, which was classified in the genus *Alysiella* and significantly associated with reduced refined grain servings. Examples of the opposite occurrence are also present, with genera such as *Mycoplasma* being associated with age, but no single ASV for this association could be identified.

We further validated our differential abundance analysis using our smaller validation data set and found 8/17 genera associated with sex, 8/16 genera associated with fat-free mass, 5/15 genera associated with height, and 3/11 genera associated with age were recoverable (Fig. S5A). Additionally, the negative association between *Prevotella* 2 and waist-hip ratio was also verified within this data set. Furthermore, several associations between ASVs and features such as sex (5/14), height (4/12), fat-free mass (2/3), and sleeping light exposure (1/2) were also found within this smaller validation data set (Fig. S5B). All significant effect sizes that were recovered in the validation data set except for one, between sleeping light exposure and ASV-d4746 *Streptococcus*, remained in the same direction as the original cohort, indicating relationships that were robust to sample choice.

10.1128/mSphere.00451-20.5 FIG S5 Validation of genera and ASV association in a smaller Atlantic Partnership for Tomorrow’s Health dataset (*n* = 308). Data from subjects who did not complete all questions examined in this study were used to validate previous associations identified in the larger cohort. The testing procedure was the same as that for Fig. 3; however, false discovery rate correction was not applied to *P* values, as previous evidence was already present for these taxonomic relationships. (A) Genera; (B) ASVs. Download FIG S5, TIF file, 2.2 MB. Copyright © 2020 Nearing et al. 2020 Nearing et al. This content is distributed under the terms of the Creative Commons Attribution 4.0 International license.

### Predicted microbial pathway abundances reveal multiple pathways associated with anthropometric, dietary, age, and sex features.

**FIG 5:**
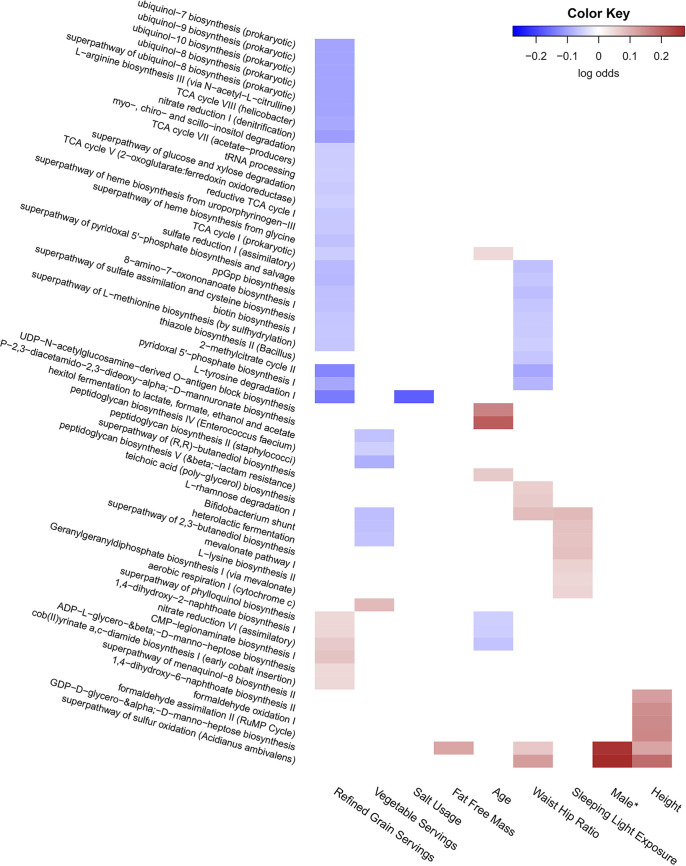
Various predicted pathway abundances are associated with features significantly associated with overall microbiome composition. Pathway abundances were predicted from 16S rRNA gene sequencing data using PICRUSt2. Predicted pathway abundances meeting an FDR of <0.05 and an effect size of |0.05| log odds were considered significant associations using the Corncob R package. Each feature’s false discovery rate was corrected separately, and each was tested to control for differences in DNA extraction and differential variability within that feature. Ordinal variables were converted into a ranked scale for testing, and all features except for sex were scaled. The asterisk indicates that sex was treated as a categorical value; therefore, the magnitude is not directly comparable to other log odd ratios.

Microbial pathway abundances were predicted using PICRUSt2 (30) to determine potential associations between pathway abundances and features previously identified to be significantly associated with differences in beta diversity. Differential analysis between features and predicted pathway abundances were done using Corncob with Benjamini-Hochberg-corrected *P* values at an alpha of 0.05, and associations with effect sizes under |0.05| log odds were filtered out. We found 9/15 features originally associated with beta diversity metrics to have at least one predicted pathway association (Fig. 5). Of these features, we found that refined grain servings had the largest number (*N* = 33) of predicted pathway associations. Of these associations, many were negatively associated with increasing refined grain intake, including various tricarboxylic acid cycle derivatives, glucose and xylose degradation, 2-methylcitrate cycle, and heme biosynthesis. Furthermore, only a smaller number of pathways were associated with increasing refined grain intake, such as phylloquinol biosynthesis and CMP-legionanimate biosynthesis (Fig. 5).

Only one pathway, aerobic respiration I (cytochrome *c*), was predicted to be associated with increasing vegetable servings, while six pathways were associated in the opposite direction. These pathways included fermentation of carbohydrates into lactate, lactic acid, ethanol, acetate, and formate as well as the biosynthesis of peptidoglycan. We found only one association with salt usage (l-tryosine degradation) and did not find any predicted associations with juice serving intake.

A number of predicted pathway abundances were also associated with various anthropometric features, with waist-hip ratio having the highest number of predicted associations (*N* = 15) and fat-free mass having only one predicted association (GDP-d-glycero-α-d-mannose-heptose biosynthesis). We also found a small number of predicted pathway abundances associated with age (*N* = 7) and sex (*N* = 2).

Validation analysis on the second smaller data set was only able to validate a minority of predicted pathway associations, many of which were associated with an individual’s waist-to-hip ratio (8/15) (Fig. S6). Only 4 of the original 33 predicted pathway associations with refined grain intake were verified within this cohort. These pathways included l-tyrosine degradation, ADP-l-glycero-α-d-manno-heptose biosynthesis, phylloquinol biosynthesis, and 1,4-dihydroxy-2-naphthoate biosynthesis. Three out of six pathways associated with height and four out of seven pathways associated with age were also found to be significant within the smaller validation data set (Fig. S6).

10.1128/mSphere.00451-20.6 FIG S6 Validation of predicted pathway abundance associations in a smaller Atlantic Partnership for Tomorrow’s Health dataset (*n* = 308). Data from subjects who did not complete all questions examined in this study were used to validate previously predicted pathway associations found in the larger cohort. Testing was done in the same manner as Fig. 4; however, false discovery correction was not applied to *P* values, as previous evidence was already present for these associations. Download FIG S6, TIF file, 2.7 MB. Copyright © 2020 Nearing et al. 2020 Nearing et al. This content is distributed under the terms of the Creative Commons Attribution 4.0 International license.

## DISCUSSION

Our analysis of 1,049 healthy (Fig. 1) individuals from Atlantic Canada revealed that much of the oral microbiome of Atlantic Canadians was made up of 11 “core” genera that belong to six different phyla (*Actinobacteria*, *Fusobacteria*, *Proteobacteria*, *Firmicutes*, *Bacteroidetes*, and *Fusobacteria*). Interestingly, some of these core genera, found in 99% of all samples, were found in relatively low abundance (<2% mean abundance), indicating that bacteria within the oral microbiome can be consistently observed with minor contributions (see Table S1 in the supplemental material). In contrast, the composition at the ASV level had only 3 ASVs being present in 99% of samples and only contributing 5.17% of the total oral microbiome composition on average. Overall, these results indicate that individuals tend to share similar genera within the oral cavity, but the species/strains shared between individuals can be highly variable. These findings are in line with previous work from the Human Microbiome Project that found the oral microbiome to be relatively similar between individuals at the genus level (1).

10.1128/mSphere.00451-20.7 TABLE S1 Mean relative abundance of the 11 genera found in 99% of all healthy oral microbiome samples. Genera abundance tables were filtered to only retain genera found in 99% of all saliva samples within the study. Both the mean relative abundance and standard deviations of relative abundance for these genera were then calculated. Download Table S1, DOCX file, 0.01 MB. Copyright © 2020 Nearing et al. 2020 Nearing et al. This content is distributed under the terms of the Creative Commons Attribution 4.0 International license.

We found that various anthropometric and lifestyle features were significantly associated with overall oral microbiome composition; however, they explained only a small amount of total oral microbiome variance while controlling for DNA extraction batch (5.87 to 7.00%) (Tables S2 and S3). We found that fat-free mass explained the largest amount of variance (0.6 to 0.9%) (Fig. 3A and B) of all biological features. While this feature had many differentially abundant genera and ASVs associated with it, we were unable to recover any of them after controlling for differences in sex. This indicates that these associations could be driven by sex and not underlying fat mass; however, we were also unable to recover many relationships between sex and taxonomic abundance while controlling for fat-free mass, indicating that both of these factors significantly confound the other. However, despite these issues there is previous evidence to suggest that some bacteria are related to differences in body size. A study in children found reduced abundance of *Veillonella*, *Prevotella*, *Selenomonas*, and *Streptococcus* in obese children (31). Interestingly, in our adult population we found similar trends, with members of the *Veillonella* family being positively associated with increasing fat-free mass and members of the *Prevotella* genus also being linked with higher fat-free mass. Another publication on the Southern Community Cohort Study found that both *Granulicatella* and *Gemella* were associated with obesity (27), which we also found within our cohort at both the genus and ASV level. One interesting result from our study was our inability to identify any genera or ASVs linked to BMI despite numerous relationships between anthropometric measurements being identified. These results indicate that future studies should include sex and other measurements of body composition, such as lean body mass, when looking at relationships between the microbiome and obesity.

We found two genera, Defluvittaleaceae UCG-011 and an uncultured genus from *Veillonellaceae*, that were strongly associated with being male (Fig. 4A). However, neither of these associations was recovered in our validation cohort, indicating that they could either be false positives or require a larger sample size to recover due to their low mean relative abundance (0.0042% and 0.063%, respectively). Despite this, we were still able to recover eight genus-level associations in our validation data set (Fig. S5A); however, only a few of these associations match those that were previously reported. Renson et al. found two genera, *Lactobacillus* and *Actinobacillus*, to be higher in males, which we did not find in our study (21). This could have been due to multiple differences, including sampling procedures, systemic protocol bias, or the compositional nature of microbiome data (32). Raju et al. found that there was a high relative abundance of *Haemophilus* in females, which we also found in our study; however, they also found *Oribacterium* to be increased in females, which was opposite from what was found in this study (31). Differences between these studies and ours can be attributed in part to differences in sample collection procedure and sequencing primers used, highlighting the technical biases in the field (33).

We were unable to recover any taxonomic relationships between dietary features within our validation data set; however, refined grain servings per day had one of the largest impacts on overall oral microbiome composition and microbial pathway potential in our initial analysis. During this initial analysis, we found that bacteria from four genera, *Bergeyella*, *Parvimonas*, *Veillonella*, and *Neisseria*, decreased in relative abundance with increasing refined grain intake (Fig. 4). Interestingly, refined grain intake had a strong association with inflammatory bowel disease (IBD) in a previous analysis of this cohort (34), and alterations in the oral microbiome have been linked to IBD in the past (35). Work by Said et al. found multiple genera in differential abundance between individuals with and without IBD, including the increased presence of *Veillonella* in IBD patients (14), which we found to be linked positively with refined grain intake.

We found that of all features significantly associated with overall oral microbiome composition, refined grain intake had the largest number of predicted pathway associations (Fig. 5). Many of these pathways were related to metabolic functions and the biosynthesis of various cofactors and metabolic building blocks, indicating a shift in metabolic potential within the microbial community. This shift is not surprising given that differing levels of refined grain intake could impact the availability of various carbohydrates to oral microbiota. However, it should be noted that only a small number of these pathway associations were verified within our small validation data set.

Other dietary factors we found linked to overall oral microbiome composition in our original analysis include both juice servings and vegetable servings. However, we were only able to find a small number of genera, ASVs, and predicted pathway abundances linked to vegetable serving intake. We found a number of fermentation pathways were predicted to be associated with reduced vegetable intake, indicating a shift in anaerobic activity. While we found a small number of taxonomic associations with juice serving intake, we found no predicted pathway associations. Furthermore, we were unable to recover any taxonomic or pathway associations for both vegetable intake or juice serving intake in our validation data set, indicating the possibility of a false positive or the requirement of a large sample size to see these effects. Previous work within the field has found conflicting evidence on the role of diet impacting oral microbiome composition and may be reflective of different dietary assessment methods.

Looking at all features that were significantly associated with oral microbiome composition together in a single model, we were only able to explain a small portion of the total variance between samples (5.87 to 7.00%). This indicates that while many of these features are significantly related to microbial composition, each one by itself tends to cause only small shifts in overall microbial composition. Furthermore, a majority of the variance accounted for was due to differences in DNA extraction date. This shows that slight technical variations, such as the time when DNA extraction was done, can have large impacts on sample composition, emphasizing the need to control for these technical variations during large population-based studies.

One large limitation to our study was our lack of detailed dental history information from participants. While we did record how recently each individual last visited the dentist, we were unable to retrieve detailed information on dental health, which has been found to have dramatic impacts on oral microbiome composition (17). Furthermore, our study was also unable to capture potential variance that could have been attributed to the time of sampling. Various studies have shown that oral microbiome composition can vary with regard to collection time due to events such as teeth brushing and eating throughout the day (36). These could explain some of the missing variation that was not accounted for in our study; however, it is unlikely to explain all 93.00%, indicating we are still missing a suitable amount of information on what determines an individual’s oral microbiome composition.

In conclusion, our study indicates that the healthy oral microbiome is relatively similar between individuals at the genus level and is impacted very little by any one factor. Future studies that attempt to identify oral microbial biomarkers associated with disease may be encouraged by the lack of major confounding variables and may be justified in controlling only for sex, body composition, oral health, and basic dietary information.

## MATERIALS AND METHODS

### Study design and population.

The current study includes the analysis of saliva samples from the Atlantic Partnership for Tomorrow’s Health (PATH) study. Atlantic PATH is part of the Canadian Partnership for Tomorrow’s Health (CanPath) project, a pan-Canadian prospective cohort study examining the influence of environmental, genetic, and lifestyle factors on the development of chronic disease (37). The applicable provincial and regional ethics boards approved the study protocol, and all participants provided written informed consent prior to participation. The primary inclusion criteria were that participants were aged 30 to 74 years at the time of recruitment and a resident in one of the Atlantic Canadian provinces (Nova Scotia, New Brunswick, Prince Edward Island, and Newfoundland and Labrador). Recruitment and baseline data for all participating regions were collected between 2000 and 2019. Details on participant recruitment and a descriptive cohort profile have been published elsewhere (37). The questionnaire included sociodemographic information, health information, behaviors, environmental factors, and self-reported anthropometric information. Participants also had anthropometric measures (height, weight, waist and hip circumferences, body composition, blood pressure, grip strength, and resting heart rate) and biological samples (blood, urine, saliva, and toenails) collected. Approximately 9,000 participants in the Atlantic PATH cohort provided a saliva sample. Participants were instructed to refrain from eating, smoking, or chewing gum for at least 30 min prior to oral specimen collection. Oral saliva specimens were collected during normal clinic hours, 9:00 a.m. to 7:00 p.m., after completion of the approximately 1-h interview and registration process. Oral samples (3 ml) were collected in sterile 50-ml conical tubes after rinsing with water. Samples were stored at 4°C and batch shipped on ice to the central processing facility at the QEII Health Sciences Centre in Halifax, Nova Scotia. Samples were processed within 24 h of collection, aliquoted into cryovials, and stored at −80°C until analysis.

The current analysis includes a total of 1,214 saliva samples from healthy Atlantic Canadians living within the provinces of Nova Scotia, New Brunswick, and Prince Edward Island. Based on self-reported data, participants were defined as healthy if they had not been diagnosed with any of the following conditions: hypertension, myocardial infarction, stroke, asthma, chronic obstructive pulmonary disease, major depression, diabetes, inflammatory bowel disease, irritable bowel syndrome, chronic bronchitis, emphysema, liver cirrhosis, chronic hepatitis, dermatologic disease (psoriasis and eczema), multiple sclerosis, arthritis, lupus, osteoporosis, and cancer. A total of 165 of these samples were removed due to insufficient sequencing depth, and of the remaining 1,049 samples, an additional 308 were removed due to incomplete answering of the 41 variables examined in this study. These 308 samples were then used in validation analysis (details below) to confirm findings within the larger 741-participant data set.

### Sociodemographic, lifestyle, and anthropometric variables.

Questionnaires were used to collect sociodemographic and lifestyle variables. Self-reported variables included age, sex, education level, household income, rural/urban status, province, dental visits, sleep patterns, alcohol consumption, smoking status, and dietary variables, such as food avoidance, the use of specific types of fat/oil, artificial sweetener usage, the frequency of dessert, soda drinks, soy/fish sauce, salt seasoning, and fast food, as well as servings of vegetables, fruit, juice, whole grains, refined grains, dairy products, eggs, fish, tofu, beans, and nuts/seeds. Anthropometric measures were collected by trained personnel in assessment centers. Waist and hip circumferences were measured using Lufin steel tape. Height was measured by a Seca stadiometer. Height and weight measures were used to calculate body mass index (BMI; weight, in kilograms, divided by height, in meters squared). Body weight, fat mass, and fat-free mass were measured using the Tanita bioelectrical impedance device (Tanita BC-418; Tanita Corporation of America Inc., Arlington Heights, IL). Table 1 lists all variables that were used for analysis.

### Oral microbiome 16S rRNA sequencing.

Frozen saliva samples were thawed at room temperature and aliquoted into 96-well plates. DNA from samples was then extracted using a QIAamp 96 PowerFecal QIAcube HT kit by following the manufacturer's instructions using a TissueLyser II and the addition of Proteinase K. Sequencing of the 16S rRNA gene was performed by the Integrated Microbiome Resource at Dalhousie University. The V4-V5 region was amplified from extracted DNA in a PCR using 16S rRNA gene V4-V5 fusion primers (515FB–926R) (38) and high-fidelity Phusion polymerase. Amplified DNA concentrations were then normalized and pooled to be sequenced on an Illumina MiSeq. The sequencing of samples was conducted over 6 Illumina MiSeq runs producing 300-bp paired-end reads.

### 16S rRNA gene sequence processing.

Primers were removed from paired-end 300-bp sequences using cutadapt (39). Primer-free reads were then stitched together using the QIIME2 (v. QIIME2-2018.8) (40) VSEARCH (41) join-pairs plugin. Stitched reads were then filtered using the QIIME2 plugin q-score-joined using the default parameters. Quality filtered reads were then input into the QIIME2 plugin Deblur (42) to produce amplicon sequence variants (ASVs). Trim length was 360 bp, and the minimum number of reads required to pass filtering was set to 1. Amplicon sequence variants that were found in an abundance of less than 0.1% of the mean sample depth (18) were then removed from analysis. This is to keep in line with the approximate bleed-through rate on an Illumina MiSeq sequencer. After filtering, a total of 13,248 ASVs were recovered. Representative sequences were then placed into the Greengenes 13_8 99% (43) reference 16S rRNA tree using the QIIME2 (2019.7) fragment insertion SEPP (44, 45) plugin. Rarefaction curves were then generated using the QIIME2 alpha-rarefaction plugin, and a suitable rarefaction depth of 5,000 was chosen for diversity analysis based on when the number of newly discovered ASVs came to a plateau (see Fig. S1 in the supplemental material). Representative sequences were then assigned taxonomy using a custom-trained V4-V5 16S rRNA naive Bayesian QIIME2 classifier (46) trained on the 99% Silva V132 database (47).

### Oral microbiome composition analysis.

Taxonomic composition tables were generated using the QIIME2 taxa plugin and collapsed at the genus level. All samples over 5,000 reads in depth (1,049) were subsampled to a depth of 5,000 reads each, and taxa that contributed less than a mean relative abundance of 1% were grouped together in the “Other” category. The composition stacked bar chart was then generated in R using ggplot2 (48), and the *x* axis was ordered based on the PC1 weighted Unifrac coordinates of each sample.

### Core oral microbiome analysis.

Taxonomic tables subsampled previously at 5,000 reads were collapsed at the genus and ASV level using QIIME2. To examine the mean relative abundance explained by genera/ASVs at different prevalence levels, we removed genera/ASVs that were not present in various numbers of samples (5 to 99%). After removal of these genera/ASVs, the remaining total mean relative abundance of all genera/ASVs that passed the filtering parameter was calculated.

### Oral microbiome alpha diversity analysis.

Alpha diversity metrics were generated using QIIME2 (v2019.7) and the previously generated tree containing both representative sequences and reference sequences. All samples were subsampled to a depth of 5,000 reads. Association between four different alpha diversity metrics (Faith’s phylogenetic diversity [PD], Shannon diversity, evenness, and number of ASVs) was then tested using general linear models while controlling for DNA extraction. A base model containing only DNA extraction as a covariate and a testing model containing DNA extraction and the covariate of interest were then compared using an ANOVA, and *P* values were recorded. Recorded *P* values were then corrected for false discovery (Benjamini and Hochberg [49]) with a chosen alpha of *q* < 0.1.

### Oral microbiome beta diversity analysis.

Beta diversity metrics were generated using QIIME2 and the previously generated phylogeny. All sequences were subsampled to a depth of 5,000 reads based on the plateauing stage of rarefaction plots (Fig. S1). Associations between two different beta diversity metrics (weighted UniFrac distance and Bray-Curtis dissimilarity) were then tested using a PERMANOVA (adonis2 function in Vegan [50]) while controlling for DNA extraction. Marginal *P* values were then corrected for false discovery (Benjamini and Hochberg), and an alpha value of *q* < 0.1 was chosen. Significant features from univariate analysis were then included in a single multivariate model that underwent backwards covariate selection, where each covariation with the highest *P* value was removed from the model until all features were found to be significant (*P* < 0.05). Additional testing using adonis2 on fat-free mass and height were done while controlling for both sex and DNA extraction. Finally, overall relationships between taxa, metadata, and samples were visualized with a redundancy analysis triplot. This plot was constructed using the rda function within the vegan R package. Within this function, nonrarified center-log-ratio genera count tables were filtered for features with at least 10% prevalence and then used as the response variable within the redundancy analysis (RDA) model. Each feature previously associated with either weighted UniFrac or Bray-Curtis dissimilarity profiles were input as explanatory variables within the RDA model. The significance of the RDA model was checked using the function anova.cca within the vegan R package. Finally, visualization of the resulting RDA model was done with the R package ggord (51) using symmetrical species and site scaling.

### Differential abundance analysis.

Differential abundance analysis was conducted using the Corncob (52) (v 0.1.0) and Phyloseq (53) R packages. A genus-level taxonomic table was generated using QIIME2 (2019.7), and genera that were not found in at least 10% of samples were removed. The 15 covariates that were found to be significantly associated with either weighted UniFrac or Bray-Curtis dissimilarities were chosen for testing. The testing of each covariate was done using the “differentialtest” function in the Corncob package while controlling for differences in DNA extraction and differential variability across DNA extraction and the covariate of interest. Heatmaps were then constructed containing any genera/ASVs that were significantly associated with at least one of the covariates that were tested.

### Prediction of microbial pathway abundances using Picrust2.

Amplicon sequence variant abundance tables were rarified at a depth of 5,000 reads and input into the picrust2_pipeline.py script to generate predicted microbial pathway abundances. MetaCyc pathway identifiers were then mapped to their respective pathway names using the picrust2 add_descripition.py script. Differential abundance analysis of predicted pathway abundances using the R package Corncob was done in the same manner as that previously explained for taxonomic data. Only features that were found to be significantly associated with weighted UniFrac or Bray-Curtis dissimilarities were tested. DNA extraction batch and differential variability within the tested feature were controlled for as previously described, and *P* values were corrected using Benjamini-Hochberg false discovery correction (49). An alpha value of 0.05 was chosen for corrected *P* values, and pathways with an effect size lower than |0.05| log odds were filtered out.

### Validation analysis.

A total of 308 subjects had not completely answered all 41 metadata variables of interest and, therefore, were removed from the original analysis. This smaller data set was used to test our previous results by removing samples during testing of each covariate that had not answered that question on the questionnaire. Both beta diversity analysis and differential abundance analysis on taxa and pathways were carried out in the same manner as that previously explained. Both beta diversity metrics using PERMANOVA tests and differential abundance analysis using Corncob were done in a univariate fashion while also controlling for DNA extraction batch. Furthermore, only features/taxa that were originally identified as being significantly associated with oral microbiome composition in our initial cohort were tested. As there was previous evidence that these features were associated with that covariate/metric, *P* values were not corrected for false discovery but an alpha value of 0.05 was chosen. Furthermore, to keep with the original pathway analysis, only pathways that had an effect size of |0.05| log odds in the discovery cohort were tested for differential abundance in the validation cohort.

### Random forest model training and validation.

Nonrarified ASV abundances were converted into relative abundances and used to train random forest classification and regression models for each feature that was significantly associated with either weighted UniFrac or Bray-Curtis dissimilarities. An optimal mtry parameter was chosen using 3-fold repeated cross validation within the caret R package (54). Trained models for each feature were then validated on the holdout validation data set to determine model performance. Model performance for classification was visualized using the PRROC R package (55), and *R*^2^ performance of regression models was determined using the postResample function within the caret R package.

### Data availability and materials.

All sequencing data have been uploaded to the European Nucleotide Archive and are available under the accession number PRJEB38175. Code used to analyze all data is available at https://github.com/nearinj/Nearing_et_al_2020_Oral_Microbiome. Deidentified metadata used in this project can be accessed by contacting the Atlantic Partnership for Tomorrow’s Health project.
