# Supplementary figures and images for: Assessing the Variation within the Oral Microbiome of Healthy Adults
Source: mSphere. 2020 Sep 30;5(5):e00451-20. doi: 10.1128/mSphere.00451-20 (PMC7529435; doi:10.1128/mSphere.00451-20)

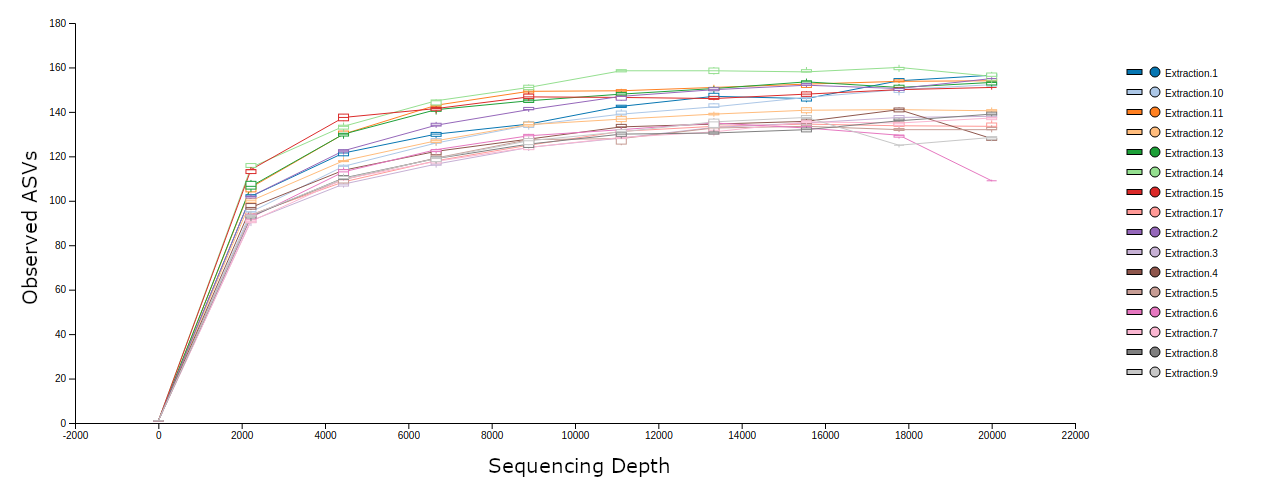

Supplement: FIG S1 [file mSphere.00451-20-sf001.tif]
