# Supplementary material for: Assessing the Variation within the Oral Microbiome of Healthy Adults
Source: mSphere. 2020 Sep 30;5(5):e00451-20. doi: 10.1128/mSphere.00451-20 (PMC7529435; doi:10.1128/mSphere.00451-20)
Supplement: TABLE S2 [file mSphere.00451-20-st002.docx]

| Feature | P-value | R^2^ |
| --- | --- | --- |
| DNA Extraction Number | 0.000999 | 0.04177 |
| Age | 0.005994 | 0.00575 |
| Refined Grain Servings | 0.003996 | 0.00566 |
| Salt Seasoning | 0.030969 | 0.00373 |
| Fat Free Mass | 0.001998 | 0.00860 |
| Sleeping Light Exposure | 0.016983 | 0.00439 |
| Total: |  | 0.06987 |
