# Supplementary material for: Assessing the Variation within the Oral Microbiome of Healthy Adults
Source: mSphere. 2020 Sep 30;5(5):e00451-20. doi: 10.1128/mSphere.00451-20 (PMC7529435; doi:10.1128/mSphere.00451-20)
Supplement: TABLE S3 [file mSphere.00451-20-st003.docx]

| Feature | P-Value | R^2^ |
| --- | --- | --- |
| DNA Extraction | 0.000999 | 0.03270 |
| Age | 0.010989 | 0.00311 |
| Refined Grain Servings | 0.003996 | 0.00396 |
| Vegetable Servings | 0.040959 | 0.00248 |
| Fat Free Mass | 0.001998 | 0.00478 |
| Last Dental Visit | 0.000999 | 0.00412 |
| Sleeping Light Exposure | 0.014985 | 0.00296 |
| Juice Servings | 0.047952 | 0.00237 |
| Total |  | 0.05870 |
