# Supplementary material for: Assessing the Variation within the Oral Microbiome of Healthy Adults
Source: mSphere. 2020 Sep 30;5(5):e00451-20. doi: 10.1128/mSphere.00451-20 (PMC7529435; doi:10.1128/mSphere.00451-20)
Supplement: TABLE S1 [file mSphere.00451-20-st001.docx]

| Taxa | Mean relative abundance | Standard Deviation of relative abundance |
| --- | --- | --- |
| *Actinomyces* | 0.01214871 | 0.009861735 |
| *Prevotella* | 0.03014280 | 0.021971701 |
| *Prevotella 7* | 0.11549857 | 0.079127940 |
| *Gemella* | 0.01402536 | 0.014607749 |
| *Granulicatella* | 0.01666749 | 0.014041083 |
| *Streptococcus* | 0.11855424 | 0.082780012 |
| *Veillonella* | 0.21489952 | 0.123237741 |
| *Fusobacterium* | 0.03727722 | 0.032857930 |
| *Leptotrichia* | 0.01567703 | 0.018765034 |
| *Neisseria* | 0.13039523 | 0.129008762 |
| *Haemophilus* | 0.07298227 | 0.057756994 |
